# 114. Long-term Protection Against Herpes Zoster (HZ) by the Adjuvanted Recombinant Zoster Vaccine (RZV): Interim Efficacy, Immuno and Safety Results at Approximately 10 Years after Initial Vaccination

**DOI:** 10.1093/ofid/ofac492.192

**Published:** 2022-12-15

**Authors:** Ana Strezova, Javier Diez-Domingo, Kamal Al Shawafi, Juan Carlos Tinoco, Meng Shi, Paola Pirrotta, Agnes Mwakingwe-Omari

**Affiliations:** GSK, Rixensart, Brabant Wallon, Belgium; FISABIO Fundación para el Fomento Investigación Sanitaria y Biomédica de la Comunitat Valenciana, Valencia, Comunidad Valenciana, Spain; Modis c/o GSK, Wavre, Brabant Wallon, Belgium; Hospital General de Durango, Durango, Durango, Mexico; GSK, Rixensart, Brabant Wallon, Belgium; GSK, Wavre, Brabant Wallon, Belgium; GSK, Rixensart, Brabant Wallon, Belgium

## Abstract

**Background:**

For the first time, we present data describing vaccine efficacy (VE), immunogenicity persistence and safety up to approximately 10 years after primary vaccination against HZ with RZV. We have previously shown that RZV demonstrated high VE against HZ in adults ≥ 50 years of age (YOA) participating in 2 phase 3 clinical trials (ZOE-50, NCT01165177 and ZOE-70, NCT01165229), and VE persisted up to year (Y) 2 in the interim analysis of the extension study (ZOSTER-049, NCT02723773). Here we describe the interim analysis for Y4 of the extension study. Understanding the persistence of VE and long-term protection against HZ can help to optimize the use of RZV in adults ≥ 50 YOA.

**Methods:**

The study design is detailed in Figure 1. Primary objective included VE against HZ over the ZOSTER-049 study. Secondary objectives included VE against HZ from 1 month post-dose 2 in the ZOE-50/-70 studies until the end of Y4 of ZOSTER-049 (Y10 after vaccination), persistence of vaccine-induced humoral immunogenicity (HI) in terms of anti-glycoprotein E (gE) antibody and cell-mediated immune (CMI) response in terms of frequency of gE-specific CD4[2+] T-cells and safety. VE analysis for ZOSTER-049 used historical control estimates from the ZOE-50/-70 placebo groups.

**Figure:**
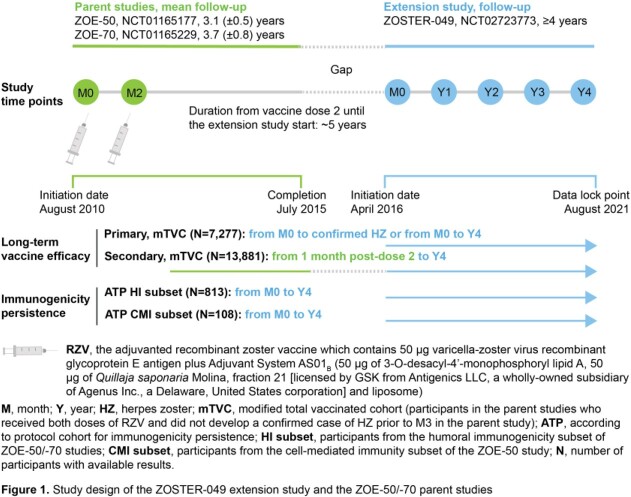

**Results:**

In ZOSTER-049, 7,413 participants were enrolled and 7,277 were included in VE analysis (Figure 2). During 4 years of follow-up in ZOSTER-049, overall VE against HZ was 81.6% (95% confidence interval [CI]: 75.2–86.6). From 1 month post-dose 2 in ZOE-50/-70 until Y4 of ZOSTER-049, the overall VE was 89.0% (95% CI: 85.6–91.3). In ZOSTER-049, anti-gE antibody concentrations persisted > 5 times above pre-vaccination up to Y10 after vaccination (Figure 3A). The frequency of gE-specific CD4[2+] T-cells remained above pre-vaccination from Y6 to Y10 after vaccination (until the end of Y4 of ZOSTER-049) (Figure 3B). No safety signals were identified until the end of Y4 of ZOSTER-049.

**Figure:**
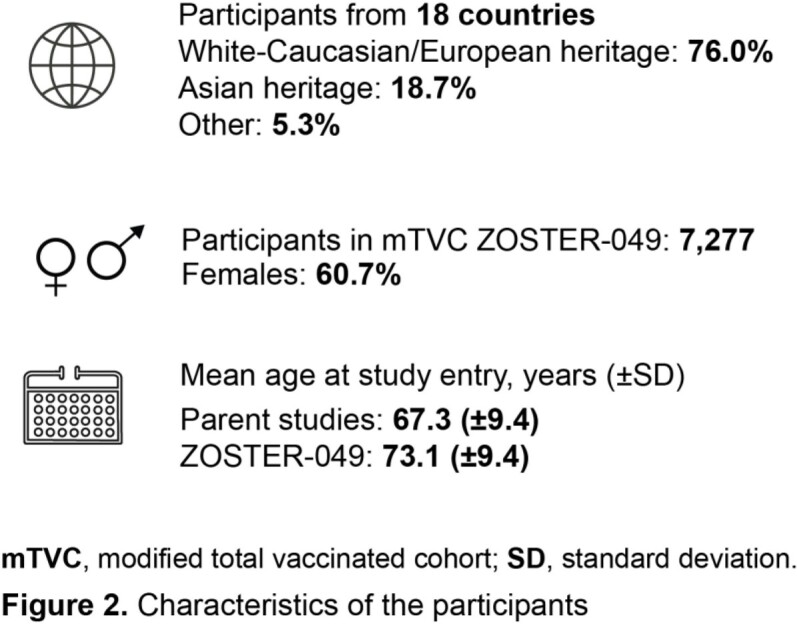

**Figure:**
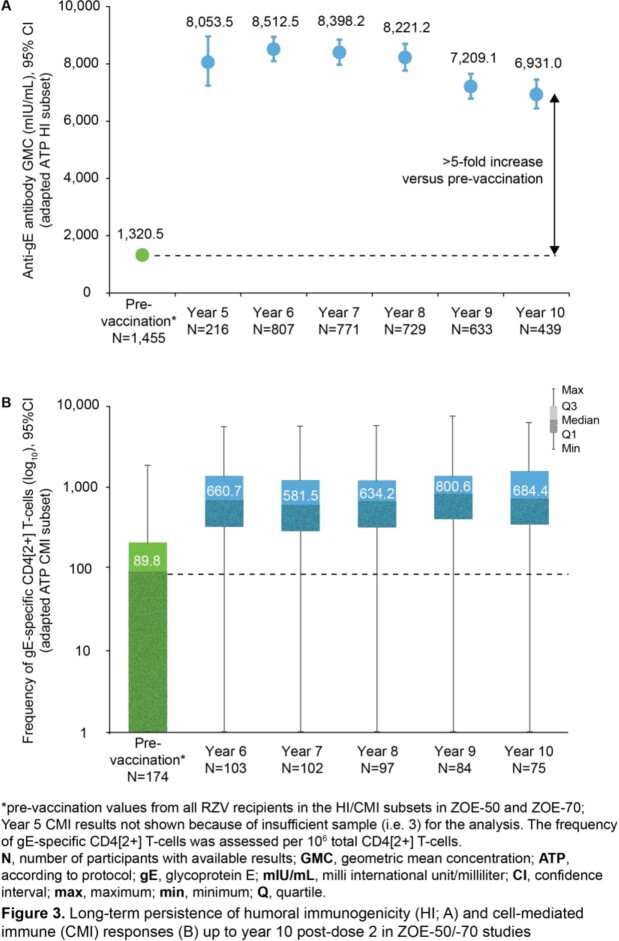

**Conclusion:**

Efficacy against HZ and immune responses to RZV remained high until the end of the observation period for this Y4 interim analysis suggesting that the clinical benefit of the RZV in adults ≥ 50 YOA is sustained for at least 10 years after vaccination. RZV safety profile remained clinically acceptable.

**Funding:**

GlaxoSmithKline Biologicals SA

**Disclosures:**

**Ana Strezova, MD, MSc**, GSK: I am an employee of GSK group of companies and hold shares from GSK group of companies as part of my employee remuneration **Javier Diez-Domingo, MD, PhD**, GSK: Grant/Research Support|GSK: Honoraria|MSD: Advisor/Consultant|MSD: Grant/Research Support|MSD: Consulting fees to the author and his institution|SANOFI: Advisor/Consultant|SANOFI: Grant/Research Support|SANOFI PASTEUR: Consulting fees to the author and his institution|SEQIRUS: Honoraria|SEQIRUS: Honoraria to the author and his institution **Kamal Al Shawafi, MD**, Modis: I am an employee of Modis, working on behalf of the GSK group of companies **Juan Carlos Tinoco, MD**, GSK: Grant/Research Support **Meng Shi, MS**, GSK: I am an employee of GSK group of companies **Paola Pirrotta, n/a**, GSK: I am an employee of GSK group of companies and hold shares from GSK group of companies as part of my employee remuneration **Agnes Mwakingwe-Omari, MD/PhD**, GSK: I am an employee of GSK group of companies.

